# From early relational experiences to non-suicidal self-injury in anorexia and bulimia nervosa: a structural equation model unraveling the role of impairments in interoception

**DOI:** 10.1007/s40519-024-01651-x

**Published:** 2024-03-25

**Authors:** Lorenzo Lucherini Angeletti, Emanuele Cassioli, Livio Tarchi, Cristiano Dani, Marco Faldi, Rachele Martini, Valdo Ricca, Giovanni Castellini, Eleonora Rossi

**Affiliations:** 1 Psychiatry Unit, Department of Health Sciences, University of Florence, Largo Brambilla, 3, 50134 Florence, Italy; 2 The Royal’s Institute of Mental Health Research & University of Ottawa, Ottawa, ON Canada

**Keywords:** Non-suicidal self-injury, Anorexia nervosa, Bulimia nervosa, Interoception, Early relational experiences, Emotional dysregulation, Dissociation

## Abstract

**Purpose:**

Individuals with anorexia nervosa (AN) and bulimia nervosa (BN) frequently exhibit Non-Suicidal Self-Injury (NSSI), yet their co-occurrence is still unclear. To address this issue, the aim of this study was to elucidate the role of impairments in interoception in explaining the NSSI phenomenon in AN and BN, providing an explanatory model that considers distal (insecure attachment/IA and traumatic childhood experiences/TCEs) and proximal (dissociation and emotional dysregulation) risk factors for NSSI.

**Method:**

130 patients with AN and BN were enrolled and administered self-report questionnaires to assess the intensity of NSSI behaviors, interoceptive deficits, IA, TCEs, emotional dysregulation and dissociative symptoms.

**Results:**

Results from structural equation modeling revealed that impairments in interoception acted as crucial mediators between early negative relational experiences and factors that contribute to NSSI in AN and BN, particularly emotional dysregulation and dissociation. Precisely, both aspects of IA (anxiety and avoidance) and various forms of TCEs significantly exacerbated interoceptive deficits, which in turn are associated to the emergence of NSSI behaviors through the increase in levels of dissociation and emotional dysregulation.

**Conclusions:**

The proposed model provided a novel explanation of the occurrence of NSSIs in patients with AN and BN by accounting for the significance of interoception.

*Level of evidence*: Level V–Cross-sectional observational study.

**Supplementary Information:**

The online version contains supplementary material available at 10.1007/s40519-024-01651-x.

## Introduction

Non-suicidal self-injury (NSSI) is the intentional and direct harm to one's own body without suicidal intent [1]. NSSI is a prevalent behavior among various psychopathologies, including Eating Disorders (EDs) [2]. Research suggested that the incidence of NSSI is particularly high among individuals with EDs, with rates of 23.2% in individuals with anorexia nervosa (AN) of the restrictive type, 42% in those with AN of the binge-purge subtype, and 37% in individuals diagnosed with bulimia nervosa (BN) [3]. This is a significant concern due to the association of NSSI with several adverse health outcomes, such as an increased risk of suicide, impaired social and interpersonal functioning, and greater severity of ED symptoms [4, 5]. Reflecting this concern, the ED population showed an increased prevalence of NSSI, suicidal ideation and attempts compared with the general population [6]. Moreover, individuals with EDs who engage in NSSI exhibited greater levels of depression, anxiety, and stress compared to those who do not engage in NSSI, suggesting that NSSI might function as a coping mechanism for emotional distress [7, 8]. However, the relationship between NSSI and EDs seems to be more profoundly intertwined.

In a recent study exploring NSSI, findings indicated that a notable portion (30%) of individuals engaging in NSSIs also exhibited symptoms of self-injurious disordered eating. Even though the majority of these individuals did not meet the criteria for an ED diagnosis, they utilized symptoms related to restrictive eating and binge-eating/purging behaviors as a form of self-injury. This subgroup of individuals demonstrated heightened clinical severity at baseline, as evidenced by higher levels of general psychopathology, lower quality of life, greater functional impairment, as well as more clinically severe NSSI [9]. Accordingly, the co-occurrence of both NSSI and EDs was positively associated with elevated severity levels for each condition [10, 11].

In the context of EDs, NSSI has been consistently associated with a range of factors that contribute to its occurrence and maintenance. Interestingly, a conceptual model of the potential factors underlying the connection between NSSI and EDs has been proposed in recent decades [2, 12]. In this model, the presence of specific distal factors facilitated the development of proximal factors that are often associated with NSSI. Precisely, the influence of distal risk factors (including traumatic childhood experiences/TCEs, personality traits, cultural pressures and the attachment style arising from a specific family environment) contributed to the emergence of proximal risk factors (such as impulsivity, self-critical cognitive styles or low self-esteem, a strong need for control, tendencies towards obsessive–compulsive behaviors, emotional dysregulation, and dissociative symptoms) [2, 12]. Within these risk factors, in individuals with EDs emotional dysregulation had been found to mediate the relationship between attachment difficulties and NSSI [13–15], whereas dissociation, as a sense of bodily detachment, mediated the relationship between TCEs and NSSI, resulting to be a crucial factor in explaining individual differences in NSSI among the ED population [16]. Moreover, individuals with EDs who engaged in NSSI demonstrated more severe emotional dysregulation and dissociative symptoms than those who did not engage in NSSI [13], indicating the intimate connection between emotional distress and self-destructive behaviors towards the body.

The body represents a putative common “battleground” of both NSSI and ED-specific behaviors, given that they both involve self-destructive behaviors, intimately tied to body-related concerns and distress. For example, instances of NSSI encompass both 'impulsive' behaviors, characterized by escalating tension prior to and gratification following the act, such as cutting or burning of the skin, and 'compulsive' behaviors, involving repetitive, seemingly purposeless motor actions, such as pulling of hair or picking at the skin [17, 18]. Similarly, ED psychopathology revolves around the body and its associated somatic, emotional, and cognitive features [19–23]. Individuals with EDs typically experience significant body dissatisfaction, regardless of their actual body weight or shape; they may thus engage in self-destructive behaviors towards the body aimed at achieving their perceived ideal body, such as restrictive eating, excessive exercise, or purging [24]. To a greater extent, what might have generated such detrimental behaviors towards the body may be traced back to the perception of internal body sensations, namely interoception [25, 26].

In line with this suggestion, numerous investigations have demonstrated a disrupted ability to sustain a connection with internal states among individuals with NSSI [27, 28] and those with EDs [29–31]. Intriguingly, it has been reported that the way an individual experiences one’s body can influence the proneness to NSSI [32, 33]. In accordance, studies indicated that interoceptive deficits are associated with NSSI in people with EDs [34–37], with interoceptive deficits remaining significant even after accounting for the presence of comorbid ED pathology through statistical analysis [32, 33]. Further, research highlighted that interoceptive deficits in patients with EDs not only differentiate between severe NSSI behaviors but also relate to difficulties in processing interoceptive cues, manifesting as overwhelming emotions or a sense of detachment or numbness [36–38].

The developmental roots of interoception lie in early relational experiences, such as attachment style and TCEs. Indeed, the interoceptive configuration depends on the quality of synchronization in the attachment relationship established with the caregiver [39]. Similarly, recent findings have highlighted how TCEs can affect an individual's ability to stay connected with one’s own internal states [40]. In parallel, the extent to which an individual is able to detect and interpret body signals has been considered to influence the formation of emotional experiences and their regulation [41, 42]. In this regard, interoceptive deficits appeared to be associated with greater emotional dysregulation in different mental health conditions [43], while also fostering a detachment from bodily experiences, which in turn might make it easier to engage in behaviors that harm the body (e.g., ED behaviors, NSSI) [32].

**Fig. 1:**
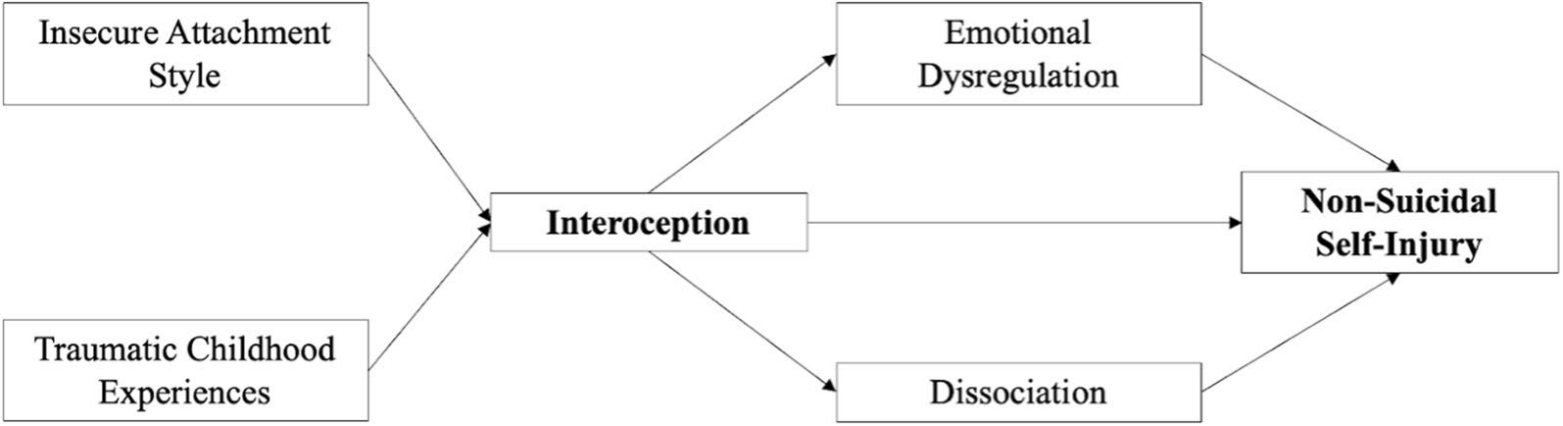
Hypothesized model of the relationship between interoception and NSSI through distal and proximal factors

These pieces of evidence suggest that considering the substantial contribution of interoception might advance the comprehension of NSSI in individuals with EDs. However, an explanatory model that disentangles the relationship between early relational experiences and NSSI, while considering the role of deficits in interoception, emotional dysregulation, and dissociation, is still lacking. It could be hypothesized that deficits in interoception may represent the link between distal risk factors, namely insecure attachment style and TCEs, and proximal risk factors for NSSI in individuals with EDs, namely emotional dysregulation and dissociation (Fig. 1). The main objective of this study was to test this hypothesis using the adoption of a structural equation modeling (SEM) approach.

## Materials and methods

### Participants

During the initial outpatient evaluation at Florence University Hospital’s EDs Clinic, between December 2021 and November 2023 a sample group of 130 female patients suffering from EDs was recruited. The selection process adhered to specific inclusion criteria, including: (i) being of female sex, (ii) age range of 18–65 years, (iii) currently diagnosed with AN or BN based on the most recent edition of the Diagnostic and Statistical Manual of Mental Disorders (DSM-5-TR) [24], and (iv) providing written informed consent for study participation and publication. Individuals who met any of the following conditions—illiteracy, intellectual disability, presence of psychotic symptoms, or being in a manic state at the time of enrollment—were excluded from the study. The study was approved from Ethics Committee of the Institution.

### Measures

Demographic information, encompassing variables, e.g., sex, age, and body mass index (BMI), was gathered. Subsequently, a standardized set of questionnaires was administered to each participant as part of the research protocol:

- Experience in Close Relationship-Revised (ECR): a 12-item self-report questionnaire used to assess attachment anxiety and avoidance in adult romantic relationships. It has demonstrated good psychometric properties in terms of reliability, construct validity, and predictive validity in several cultures and languages, with Cronbach’s α coefficients ranging from 0.80 to 0.93 [44]. The Italian validation confirmed the two-factor structure of attachment anxiety and avoidance and demonstrated good internal consistency, test–retest reliability, and convergent validity with related constructs [45]. The reliability in the sample of the present study was excellent (McDonald’s ω = 0.93).
- Childhood Trauma Questionnaire (CTQ): a 28-item self-report measure developed to retrospectively assess TCEs such as neglect and abuse [46]. The CTQ evaluates severity and presence of five early adverse experiences: Physical Abuse, Emotional Abuse, Sexual Abuse, Emotional Neglect, and Physical Neglect. Elevated CTQ scores constitute important predictors of depression, dissociation, and post-traumatic stress disorder [47]. The Italian validation by Sacchi et al. [48] confirmed a five-factor model with strong concurrent validity and reliability, which the present sample also supported (McDonald’s ω = 0.95).
- Eating Disorder Inventory-3 (EDI-3): a widely used self-report questionnaire that assesses the presence and severity of psychological traits and symptomatology associated with ED [49]. It is structured on “eating disorder-specific scales” (drive for thinness, bulimia, and body dissatisfaction), “psychological trait scales” (low self-esteem, personal alienation, interpersonal insecurity, interpersonal alienation, interoceptive deficits, emotional dysregulation, perfectionism, asceticism, and maturity fears), and “composite scales” (eating concerns composite, ineffectiveness composite, interpersonal problems composite, affective problems composite, overcontrol composite, global psychological maladjustment). In the present study, only the subscale concerning interoceptive deficits was considered. This subscale consists of 9 items regarding confusion in accurately identifying and responding to emotional states. It includes a cluster of items labeled “fear of affect”, which reflects distress experienced when emotions become overwhelming or uncontrollable, and contrasts with an “affective confusion” cluster that points to challenges in accurately recognizing emotional states. The issue of confusion and distrust concerning emotional and physical responses is frequently highlighted as a significant trait in individuals who develop EDs [49]. Higher scores indicate greater deficits in interoceptive abilities. Various studies on EDs have shown that the interoceptive deficits subscale of the EDI-3 is a reliable index of the perception of one’s own body signals in this population [36, 37]. The Italian version found good internal consistency for most of the subscales, with Cronbach’s α values ranging from 0.70 to 0.93 [50]. The questionnaire showed excellent reliability in the present sample (McDonald’s ω = 0.96), as well as the interoceptive deficits subscale (McDonald’s ω = 0.91).
- Difficulties in Emotion Regulation Scale (DERS): a 36-items self-report questionnaire designed to assess various aspects of emotion dysregulation. The DERS total scores range from 36 to 180, with higher scores indicating greater difficulties with emotion regulation. The internal consistency of the DERS has been found to be high, with reported Cronbach’s α coefficients ranging from 0.93 to 0.96 [51]. The Italian version was translated by Sighinolfi and colleagues [52] showing Cronbach’s α coefficient for the total scale of 0.93, indicating high internal consistency [53] which was confirmed in the present sample (McDonald’s ω = 0.96).
- Dissociative Experiences Scale (DES): a self-report inventory comprising 28 items that evaluates the extent of dissociative experiences by measuring the proportion of time an individual experiences dissociative symptoms [54]. Empirical research results demonstrate that the DES has adequate reliability (ranging from 0.85 to 0.95) and convergent validity (ranging from 0.96) across both clinical and non-clinical populations. The Italian version was translated by Schimmenti [55] and showed excellent psychometric properties, including high internal consistency as confirmed in the present sample (McDonald’s ω = 0.95).
- Repetitive Non-Suicidal Self-Injury Questionnaire (R-NSSI-Q): a reliable and valid measure consisting of 15 items that can assist in identifying the risk of NSSI [56]. The R-NSSI-Q items are aligned with DSM-5-TR criteria, including the failure to resist the urge to self-injure or fear of being unable to resist (Criterion B2), increased tension before engaging in NSSI (Criterion B1), a sense of gratification and relief after self-injuring (Criterion B4), difficulty sharing the NSSI experience (Criterion C), and the repetitive nature of NSSI (not fully satisfying Criterion B3) [24]. The R-NSSI-Q has demonstrated satisfactory reliability in multiple studies [56, 57], and a score of 21 on the R-NSSI-Q was identified as an optimal cut-off for distinguishing between occasional and repetitive NSSI in adolescents. The reliability in the sample of the present study was excellent (McDonald’s ω = 0.96).

### Statistical analysis

Patients were compared for socio-demographic characteristics and study variables using age-adjusted analysis of covariance (ANCOVA). Following the transdiagnostic theory that postulates that AN and BN are characterized by the same psychopathological core [20], it was expected that the results for the investigated variables would be equivalent between the two groups resulting in consequential statistical analyses in the entire EDs sample. Subsequently, multiple regressions were performed on the merged sample to analyze the correlations between the study variables with NSSI.

The SEM technique was used to test the proposed model and the paths between variables. Specifically, the hypothesized model involved a serial mediation with four stages, in which distal risk factors (insecure attachment style/ECR and TCEs/CTQ, stage 1) contributed to the impairment of interoception (interoceptive deficits/EDI-3); the latter led to higher levels of proximal ED-related factors (emotional dysregulation/DERS and dissociative symptoms/DES, stage 2), which in turn amplified NSSI behaviors (stage 3). In the first phase, the TCEs variable was modelled as a latent variable comprising the subscales of the CTQ: emotional abuse, emotional neglect, physical abuse, physical neglect, sexual abuse. The variables of each phase were regressed on all those of the previous phases, in accordance with the serial mediation theory. To facilitate the convergence of the SEM model, the ECR, EDI-3, DERS, and R-NSSI-Q scores were divided by 10. This linear transformation does not change the underlying relationships between the variables but rather rescales the values to a smaller range, improving the computational stability of the model. The reported non-standardized effects should be interpreted with this rescaling in mind. For instance, this means that a one-unit change in the reported results corresponds to a ten-unit change in the original scale of measurement.

The scaling of the latent variable was defined using the variable-marker method. Unstandardized and standardized estimates of all parameters were calculated for both observed and latent variables (fully standardized solution). While standardized coefficients are useful for comparing the relative strength of effects within the same model, unstandardized coefficients are crucial for reproducibility and generalizability purposes. During the preliminary phase of analysis, alternative models adjusted for confounding variables (age, illness duration, BMI) were tested; however, since the fit indices worsened compared to the original model, such models were discarded.

SEM analysis was performed using the maximum likelihood estimator with robust Huber-White standard errors, scalar test statistics and robust fit measures (MLR estimator). This method produces errors, test statistics and fit measures that are robust to non-normality and can handle incomplete data. Model fit was assessed by calculating the following commonly used fit measures: Comparative Fit Index (CFI ≥ 0.95 for a good fit), Tucker-Lewis Index (TLI ≥ 0.95 for a good fit), Root Mean Square Error of Approximation (RMSEA ≤ 0.06 for a good fit), Standardized Root Mean Square Residual (SRMR ≤ 0.08 for a good fit) [58]. Finally, all possible indirect effects of negative early relational experiences on NSSIs were tested by calculating bootstrapped confidence intervals (CI) corrected for bias, with 10,000 resamples; the mediation effect was considered statistically significant if the CI did not include zero.

Analyses were performed with R statistical software v4.3.0 and the Integrated Development Environment RStudio v2023.3.1.446 [59, 60], with the help of the following libraries: dplyr, lavaan, psych [61–63].

## Results

The final sample consisted of 130 individuals affected by an ED: 79 with AN (37 restricting and 42 binge-eating/purging) and 51 with BN. The median age was 21 (interquartile range: 18–27 years), and the median duration of illness was 3 years (interquartile range: 1.5–7 years). Socio-demographic and psychopathological characteristics of the sample were reported in Table 1. The only statistically significant contrast between the two groups was observed in BMI levels, with AN exhibiting lower levels and BN showing higher levels. The remaining variables showed similarity between the two groups, who reported elevated and comparable eating psychopathology scores as observed in the EDI-3 Eating Concerns Composite score (Table 1 and Supplementary Table S1). These scores are in the 80th percentile, confirming the high severity of the conditions in our sample [49]. This data supports the existence of a shared psychopathological basis across both AN and BN groups [20], leading us to continue with subsequent analyses using the unified ED sample.

**Table 1:** Characteristics of the sample divided by group, BMI and age-adjusted

	AN (n = 79)		BN (n = 51)		F
	*Mean*	*SD*	*Mean*	*SD*	
Age	22.17	6.20	24.83	10.75	0.48
BMI	15.51	1.43	20.21	2.18	156.07***
ECR avoidance	23.79	9.34	21.90	8.32	0.86
ECR anxiety	29.95	8.07	31.08	8.49	0.01
CTQ emotional neglect	11.51	4.81	12.22	4.86	0.02
CTQ emotional abuse	9.26	5.21	9.18	4.50	0.68
CTQ sexual abuse	6.44	3.50	6.63	4.16	0.56
CTQ physical neglect	6.42	1.87	6.57	1.89	1.08
CTQ physical abuse	6.19	3.09	6.49	3.00	0.04
CTQ total score	39.82	14.73	41.10	14.79	0.30
EDI-3 eating concerns composite	56.20	18.87	63.08	20.59	0.02
EDI-3 interoceptive deficits	18.52	9.26	19.16	9.90	0.01
DES total score	2.86	1.88	3.18	2.19	0.44
DERS total score	115.87	28.51	115.43	31.32	0.79
R-NSSI-Q frequency	1.69	5.73	2.76	7.17	1.13
R-NSSI-Q first age	16.15	5.54	16.00	7.19	1.71
R-NSSI-Q total score	21.67	7.55	23.43	8.58	0.82

*BMI* body mass index, *EDI-3* eating disorder inventory, *DES* dissociative experience scale, *DERS* difficulties in emotion regulation scale, *R-NSSI-Q* repetitive non-suicidal self-injury questionnaire, *ECR* experiences in close relationships, *CTQ* childhood trauma questionnaire

^***^p < 0.001

Within the unified ED sample, regression analyses showed that R-NSSI-Q total score correlated significantly with all investigated domains, except for attachment avoidance (Table 2). NSSI showed strong and moderate associations with TCEs and anxiety attachment, respectively. It also displayed a significant relationship with interoceptive deficits, as well as showing consistent associations with total scores of dissociative symptoms and emotional dysregulation (Table 2). Moreover, the frequency of NSSI behaviors was positively associated with dissociative symptoms, emotional abuse, sexual abuse and CTQ total score (Table 2). Finally, the subscale examining the age of first onset of NSSI behaviors demonstrated no associations with the investigated domains.

**Table 2:** Correlational analysis between investigated variables and self-injury within the sample, BMI and age-adjusted

	R-NSSI-Q Total score	R-NSSI-Q Frequency	R-NSSI-Q First age
EDI-3 interoceptive deficits	0.34***	0.06	0.11
DES total score	0.56***	0.22*	− 0.06
DERS total score	0.45***	0.13	− 0.07
ECR avoidance	0.11	0.07	0.15
ECR anxiety	0.24*	− 0.08	− 0.08
CTQ emotional neglect	0.36***	0.12	0.01
CTQ emotional abuse	0.51***	0.29**	− 0.03
CTQ sexual abuse	0.40***	0.40***	− 0.14
CTQ physical neglect	0.37***	0.19	0.01
CTQ physical abuse	0.40***	0.14	− 0.05
CTQ total score	0.54***	0.31**	− 0.06

*R-NSSI-Q* repetitive non-suicidal self-injury questionnaire, *EDI-3* eating disorder inventory, *DES* dissociative experience scale, *DERS* difficulties in emotion regulation scale, *ECR* experiences in close relationships, *CTQ* childhood trauma questionnaire

^*^p < 0.05

^**^p < 0.01

^***^p < 0.001

### Structural equation modeling analysis

**Fig. 2:**
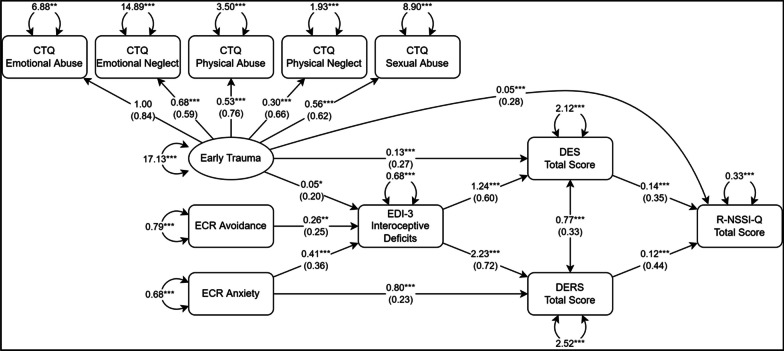
Results of the SEM analysis. Rectangles represent observed variables; circles represent latent variables. Regression effects and loadings are illustrated as single-headed arrows, together with their respective unstandardized coefficients; standardized coefficients are reported in parenthesis. *EDI-3* eating disorder inventory, *DES* dissociative experience scale, *DERS* difficulties in emotion regulation scale, *R-NSSI-Q* repetitive non-suicidal self-injury questionnaire, *ECR* experiences in close relationships, *CTQ* childhood trauma questionnaire. *p < 0.05, **p < 0.01, ***p < 0.001

The tested SEM is reported in Fig. 2. Robust fit indices demonstrated excellent model-data fit, with the following values: χ^2^ = 33.45 (p = 0.220), CFI = 0.992, TLI = 0.984, RMSEA = 0.037, SRMR = 0.050. These confirmed that the collected data were consistent with the hypothesized model.

As shown in Fig. 2, TCEs and insecure attachment styles significantly predicted higher levels of interoceptive deficits, which in turn were associated with an increased propensity for dissociation and emotion dysregulation. Furthermore, DES and DERS total scores significantly predicted higher levels of NSSI, as measured by the R-NSSI-Q Total Score (Fig. 2). Although early trauma directly predicted self-injury, ECR scores did not (Fig. 2).

Serial mediation analysis within the SEM framework confirmed that TCEs had a significant indirect effect on self-injury through the elevation in interoceptive deficits and the consequent higher levels of both dissociative symptomatology and emotion dysregulation [b_tce-id_*b_id-des_*b_des-nssi_ = 0.008, 95% CI (0.003, 0.020); b_tce-id_*b_id-ders_*b_ders-nssi_ = 0.012, 95% CI (0.003, 0.032)] (Table 3).

**Table 3:** Results of different mediation pathways in the sample

Mediation Path	Indirect effect	Bootstrapped 95% confidence interval
Early trauma → EDI-3_Interoceptive deficits_ → DES_Total score_ → R-NSSI-Q_Total score_	0.008	0.003–0.020
Early trauma → EDI-3_Interoceptive deficits_ → DERS_Total score_ → R-NSSI-Q_Total score_	0.012	0.003–0.032
Early trauma → DES_Total score_ → R-NSSI-Q_Total score_	0.018	0.007–0.041
ECR_Avoidance_ → EDI-3_Interoceptive deficits_ → DES_Total score_ → R-NSSI-Q_Total score_	0.045	0.009–0.107
ECR_Avoidance_ → EDI-3_Interoceptive deficits_ → DERS_Total score_ → R-NSSI-Q_Total score_	0.069	0.017–0.152
ECR_Anxiety_ → EDI-3_Interoceptive deficits_ → DES_Total score_ → R-NSSI-Q_Total score_	0.072	0.015–0.148
ECR_Anxiety_ → EDI-3_Interoceptive deficits_ → DERS_Total score_ → R-NSSI-Q_Total Score_	0.110	0.047–0.219
ECR_Anxiety_ → DERS_Total SCORE_ → R-NSSI-Q_Total score_	0.096	0.037–0.194
Total indirect effects		
Early trauma → R-NSSI-Q_Total score_	0.038	0.016–0.072
ECR_Avoidance_ → R-NSSI-Q_Total score_	0.115	0.032–0.227
ECR_Anxiety_ → R-NSSI-Q_Total score_	0.278	0.149–0.477

*EDI-3* eating disorder inventory, *DES* dissociative experience scale, *DERS* difficulties in emotion regulation scale, *R-NSSI-Q* repetitive non-suicidal self-injury questionnaire, *ECR* experiences in close relationships, *CTQ* childhood trauma questionnaire

Similar mediation paths were also statistically significant for insecure attachment styles, highlighting the indirect effects of both ECR Avoidance and Anxiety on the intensity of NSSI behaviors [Anxiety: b_anx-id_*b_id-ders_*b_ders-nssi_ = 0.110, 95% CI (0.047, 0.219); b_anx-id_*b_id-des_*b_des-nssi_ = 0.072, 95% CI (0.015, 0.148); Avoidance: b_av-id_*b_id-ders_*b_ders-nssi_ = 0.069, 95% CI (0.017, 0.152); b_av-id_*b_id-des_*b_des-nssi_ = 0.045, 95% CI (0.009, 0.107)] (Table 3).

Significant mediation paths were also identified through higher dissociative symptoms directly (for early trauma) and through more severe emotion dysregulation (for ECR Anxiety) [b_tce-des_*b_des-nssi_ = 0.018, 95% CI (0.007, 0.041); b_anx-ders_*b_ders-nssi_ = 0.096, 95% CI (0.037, 0.194)] (Table 3), suggesting that the mediation effect of interoceptive deficits on the association between these domains and NSSI was partial.

Taking all mediation paths into account, the total indirect effects of early trauma and insecure attachment styles on self-injury were all statistically significant (Table 3).

## Discussion

The current study sought to provide an explanatory model of NSSI in patients with EDs, investigating the role of deficits in interoception as a possible link between distal and proximal factors previously associated with NSSI in this population, including insecure attachment style and TCEs, emotional dysregulation and dissociation.

As expected, these data revealed consistent associations between NSSI and insecure attachment styles, TCEs, emotional dysregulation and dissociative symptoms confirming prior research in the field of EDs [13, 15, 16]. Additionally, NSSI exhibited a significant correlation with interoceptive deficits, in alignment with previous research investigating interoception in relation to NSSI in individuals with EDs [34–37].

**Fig. 3:**
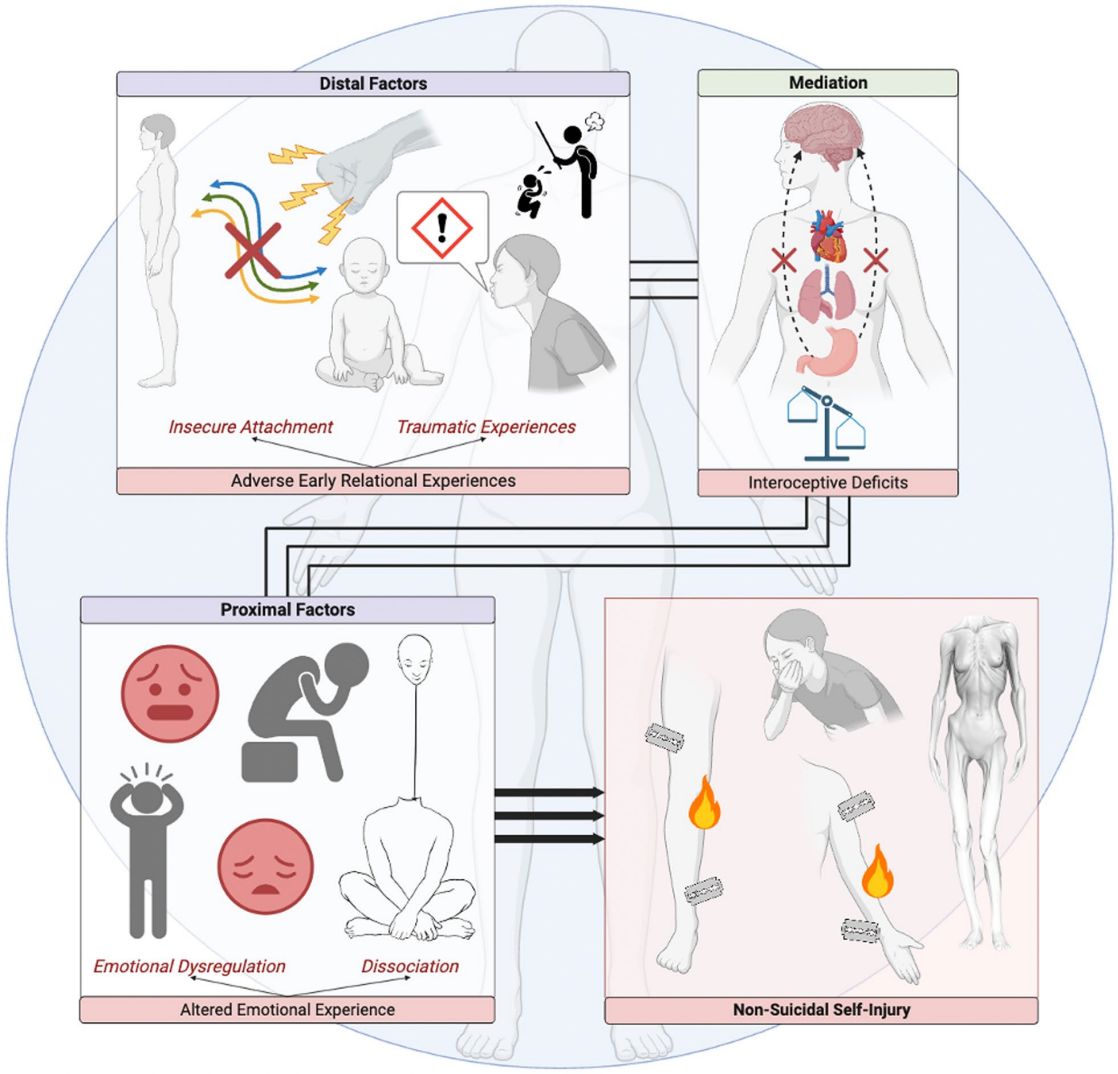
Graphic representation of the model

Regarding the explanatory model, briefly illustrated in Fig. 3, the preliminary hypothesis was substantiated (Fig. 1). Specifically, deficits in interoception were found to be key mediators in the link between early relational experiences, namely insecure attachment and TCEs, and the proximal factors commonly associated with the enactment of NSSI in patients with EDs, specifically emotional dysregulation and dissociation. Notably, among the distal factors, all the assessed facets of insecure attachment style (anxiety and avoidance) and of TCEs (from emotional to physical and sexual abuse, emotional and physical neglect) were found to play a significant role in heightening interoceptive deficits, which in turn was associated to the emergence of NSSI through the increase in levels of dissociation and emotional dysregulation (Fig. 3). Therefore, for the first time, the present model allows for a comprehensive view of the phenomenon of NSSI in patients with EDs by accounting for the significance of interoception.

Neurobiological data reported in previous studies support the proposed model about the centrality of interoception deficits in linking negative early relational experiences, emotional dysregulation, dissociation, and NSSI. Specifically, recent literature underlined modifications in the activity of the interoceptive neural network (consisting of the insula and subcortical structures) in individuals with EDs, suggesting implications at the psychopathological level [64].

This network is closely intertwined with the nature of early life primary relationships [39, 65]. In the context of attachment, a non-intentional disattunement of the caregiver, defined as “early relational trauma”, is central in determining a “disorganized interoceptive input processing” that significantly affects bodily experience [66]. For instance, if a primary caregiver fails to provide essential nourishment and emotional support, the interoceptive signals to subcortical areas and the insula may become inconsistent or absent, impeding the development of an appropriate dynamic within these limbic subcortical and cortical regions [67–71].

Similarly, early traumas appear to disrupt an individual's ability to sustain a connection with their internal states, yielding significant repercussions in later adulthood [40]. Consistently, neuroimaging studies have underscored the impact of exposure to TCEs in neural impairments in the interoceptive network, especially in the insula, medial prefrontal cortex and subcortical structures [72, 73]. Collectively, these findings emphasized how negative early relational experiences can disrupt interoceptive neural network regions (such as insula and subcortical structures) engendering a disturbance in the perception of internal states during infancy, which carries considerable implications for later adulthood.

Representing the primary cortical hub for processing visceral information and interoception, insula is thought to have a pivotal role in emotional experiences and subjective feelings [74, 75]. Indeed, the activation of the insula predicts both individual variations in interoceptive sensitivity (as the ability to accurately perceive and interpret internal bodily signals) and the reporting of negative emotional experiences, showing a correlation between these factors [76]. Additionally, functional neuroimaging studies consistently reveal insula activation when participants are exposed to emotionally arousing stimuli, such as disgusting, frightening, happy, sad, or sexual images [77].

In this context, interoceptive deficits are associated with more difficulties in identifying emotions, which might increase the risk of emotion regulation difficulties [42]. Numerous studies conducted in the general population have indicated that interoceptive deficits are connected to increased levels of alexithymia, reduced capacity to distinguish emotions in others, diminished empathy, decreased emotional responsiveness, and less ability to downregulate negative emotions [31, 78–84]. 

In addition, a recent study showed that those with a history of NSSI demonstrated significant deficits in interoception (especially in interoceptive accuracy) [85], suggesting that low interoceptive accuracy may be the biological basis for reports of ‘absent affect’, ‘detachment’ and ‘disembodiment’ in individuals who engage in NSSI [38].

Together, the present model provides an integrated and novel explanation of what has previously been shown regarding trauma and attachment as a risk factor for NSSI in patients with EDs. Within this framework, the model emphasizes the function of NSSI in EDs as essential in manipulating how the body contributes to the experience of emotions, particularly when there are deficits in interoception. Consequently, NSSI becomes an alternative way to embody an emotional experience without the need to recognize unpleasant or unreliable interoceptive cues [38]. NSSI can thus serve as a behavioral response to the negative affective states associated with eating psychopathology [86]. In addition, it has recently been hypothesized that NSSI constitutes a strategy of ‘acting on the body’ in maladaptive ways while experiencing uncertainty in bodily information, i.e., deficits in interoception [87, 88]; this may represent an attempt to reclaim the body after experiencing a sense of detachment from it.

From a clinical perspective, these data suggest that a key aspect of the therapeutic approach for people with EDs reporting NSSI could be to focus on the perception of internal states to facilitate a more adaptive emotional experience. For example, body-centered therapeutic modalities, employing bottom-up stimulation, may have the potential to help the patients to rediscover the experience of one’s own body by reintegrating emotional sensations and activations into a sense of self [89, 90]. Through a revitalization of the connection with the body, these approaches are intended to restore patients' ability to engage with their body and to rehabilitate it as a safe place to trust, thus promoting a reduction in maladaptive compensatory strategies such as NSSI.

## Strength and limits

The present study had some limitations that need to be considered. Firstly, the data relied solely on self-report questionnaires, which might have been susceptible to social desirability and self-report bias. Secondly, the recruitment of a sample comprised exclusively of women limits the generalizability of the results to the entire ED population. Thirdly, a limited sample size was enrolled, necessitating an expansion of this group to strengthen the significance of our findings. In addition, within the present model, depressive symptomatology was not taken into account. This represents a limitation, even though the evaluation of the role of depression was not the primary objective of the study. In fact, the latter was intended to introduce interoception into previously hypothesized and validated models [2, 12] by investigating the relationship between specific distal relational factors and specific proximal factors which are not strictly related to an emotional domain. Dissociative symptomatology and emotional dysregulation in fact are cross-sectional symptomatologic manifestations that are influenced by both depressive and anxious symptoms [91, 92]. Moreover, another limitation of the study is that we did not consider the duration of illness. However, we hypothesized that the latter might have a quantitative effect on severity (i.e., greater interoceptive impairments) rather than qualitative relationship between the variables under consideration (i.e., the aim of the study). Finally, caution is warranted in the generalizability of current results, and in interpreting the direction of effect solely by cross-section results here presented. Longitudinal studies are necessary to elucidate the directions of the correlations found in our study. Future studies might shed light on the impact that body-centered psychotherapies may have in decreasing the enactment of NSSIs by patients with EDs.

## What is already known on this subject?

Non-suicidal self-injury has been consistently associated with a range of factors that contribute to its occurrence and maintenance in patients with eating disorders. Precisely, specific distal factors (such as negative early relational experiences) and proximal factors (such as emotional dysregulation and dissociative symptoms) have been associated with the occurrence of NSSI. However, an explanatory model that disentangles the relationship between distal and proximal risk factors with NSSI, while considering the role of deficits in interoception, is still lacking.

## What does this study add?

This study provides an explanatory model of the relationships between distal (including traumatic childhood experiences and insecure attachment style) and proximal risk factors (including emotional dysregulation and dissociative symptoms) for non-suicidal self-injury in patients with an eating disorder, introducing deficits in interoception as a novel and essential mediator. Ultimately, this model emphasizes the importance of interoception in understanding non-suicidal self-injury phenomena in individuals with an eating disorder also suggesting new body-centered therapeutic strategies as possibly effective in patients with eating disorders reporting non-suicidal self-injury.

### Supplementary Information

Below is the link to the electronic supplementary material.

Supplementary file1 (DOCX 17 KB)

## Data Availability

Data will be made available on request.
