# Supplementary material for: From early relational experiences to non-suicidal self-injury in anorexia and bulimia nervosa: a structural equation model unraveling the role of impairments in interoception
Source: Eat Weight Disord. 2024 Mar 25;29(1):22. doi: 10.1007/s40519-024-01651-x (PMC10963569; doi:10.1007/s40519-024-01651-x)
Supplement: Supplementary file 1 — Supplementary file1 (DOCX 17 KB) [file 40519_2024_1651_MOESM1_ESM.docx]

**From Early Relational Experiences to Non-Suicidal Self-Injury in Anorexia and Bulimia Nervosa: A Structural Equation Model unraveling the Role of Impairments in Interoception**

Lorenzo Lucherini Angeletti^1,2^, Emanuele Cassioli MD^1^, Livio Tarchi MD^1^, Cristiano Dani MD^1^, Marco Faldi MD^1^, Rachele Martini^1^, Valdo Ricca MD^1^, Giovanni Castellini MD PhD^1^, Eleonora Rossi MD^1^

1. Psychiatry Unit, Department of Health Sciences, University of Florence, Largo Brambilla, 3, 50134 Florence, Italy
2. The Royal’s Institute of Mental Health Research & University of Ottawa, Ottawa, ON, Canada

**Supplementary Table 1 –** Eating Disorder Inventory (EDI-3) scores of the sample divided by group, BMI and age-adjusted. * = p<0.05.

|  | **AN (n = 79)** | **BN (n = 51)** | **F** |
| --- | --- | --- | --- |
| **EDI-3 Drive for Thinness** | 21.48 ± 7.23 | 21.64 ± 6.93 | 0.07 |
| **EDI-3 Bulimia** | 7.41 ± 8.95 | 14.10 ± 9.41 | 0.17 |
| **EDI-3 Body Dissatisfaction** | 27.31 ± 8.35 | 27.34 ± 9.20 | 0.23 |
| **EDI-3 Low Self-Esteem** | 15.08 ± 6.01 | 14.30 ± 6.09 | 0.03 |
| **EDI-3 Personal Alienation** | 13.95 ± 6.18 | 13.76 ± 7.24 | 0.01 |
| **EDI-3 Interpersonal Insecurity** | 14.51 ± 6.00 | 12.98 ± 5.95 | 1.15 |
| **EDI-3 Interpersonal Alienation** | 9.68 ± 4.50 | 10.18 ± 4.37 | 0.13 |
| **EDI-3 Interoceptive Deficits** | 18.59 ± 9.18 | 18.90 ± 9.83 | 0.02 |
| **EDI-3 Emotional Dysregulation** | 9.21 ± 6.25 | 9.92 ± 7.69 | 0.25 |
| **EDI-3 Perfectionism** | 11.57 ± 5.36 | 10.62 ± 5.17 | 0.00 |
| **EDI-3 Ascetism** | 12.17 ± 5.81 | 11.78 ± 6.60 | 0.00 |
| **EDI-3 Maturity Fears** | 15.81 ± 8.16 | 16.22 ± 7.86 | 4.15* |
| **EDI-3 Eating Concerns Composite** | 56.20 ± 18.87 | 63.08 ± 20.59 | 0.02 |
| **EDI-3 Ineffectiveness Composite** | 29.03 ± 11.50 | 28.06 ± 12.55 | 0.02 |
| **EDI-3 Interpersonal Problems Composite** | 24.19 ± 9.24 | 23.16 ± 9.26 | 0.75 |
| **EDI-3 Affective Problems Composite** | 27.80 ± 14.11 | 28.82 ± 15.77 | 0.11 |
| **EDI-3 Overcontrol Composite** | 23.75 ± 9.81 | 22.40 ± 9.67 | 0.00 |
| **EDI-3 Global Psychological Maladjustment** | 120.57 ± 39.88 | 118.66 ± 44.19 | 0.12 |
|  | | | |
